# Iodide-Mediated Etching of Gold Nanostar for the Multicolor Visual Detection of Hydrogen Peroxide

**DOI:** 10.3390/bios13060585

**Published:** 2023-05-28

**Authors:** Yunping Lai, Beirong Yu, Tianran Lin, Li Hou

**Affiliations:** School of Chemistry and Pharmaceutical Sciences, State Key Laboratory for Chemistry and Molecular Engineering of Medicinal Resources, Guangxi Normal University, Guilin 541004, China; lyp159357@stu.gxnu.edu.cn (Y.L.); yubeirong@stu.gxnu.edu.cn (B.Y.)

**Keywords:** iodide, gold nanostar, H_2_O_2_

## Abstract

A multicolor visual method for the detection of hydrogen peroxide (H_2_O_2_) was reported based on the iodide-mediated surface etching of gold nanostar (AuNS). First, AuNS was prepared by a seed-mediated method in a HEPES buffer. AuNS shows two different LSPR absorbance bands at 736 nm and 550 nm, respectively. Multicolor was generated by iodide-mediated surface etching of AuNS in the presence of H_2_O_2_. Under the optimized conditions, the absorption peak Δλ had a good linear relationship with the concentration of H_2_O_2_ with a linear range from 0.67~66.67 μmol L^−1^, and the detection limit is 0.44 μmol L^−1^. It can be used to detect residual H_2_O_2_ in tap water samples. This method offered a promising visual method for point-of-care testing of H_2_O_2_-related biomarkers.

## 1. Introduction

Hydrogen peroxide (H_2_O_2_) is widely used in fields such as the textile, chemical, environmental protection, medical, and food industries due to its ecological friendliness, strong oxidation ability, and ability to kill bacteria and bleach [1,2,3,4]. However, the direct addition of H_2_O_2_ in food production is prohibited by most countries, as residual H_2_O_2_ and its derived active substances can cause oxidative damage to human health [5,6]. Food-grade H_2_O_2_ residue is considered a “recognized safe substance” (JECFA, 2004), and according to the regulations of the Food and Agriculture Organization of the United States, the maximum concentration of H_2_O_2_ in dairy products is limited to 0.05%. In the Chinese national standard (GB53009.226-2016), the H_2_O_2_ content in food is strictly limited to less than 3 mg kg^−1^. Excessive H_2_O_2_ may cause Alzheimer’s, Parkinson’s, amyotrophic lateral sclerosis, diabetes, traumatic brain injury, ischemia/reperfusion injury, learning and memory impairment, and other diseases [7,8,9,10]. Therefore, developing a simple and effective method to monitor H_2_O_2_ concentration is of great significance.

At present, the commonly used technologies for H_2_O_2_ detection include the colorimetric method [11,12,13], electrochemical method [14,15], fluorescence method [16,17], liquid chromatography [18,19], SERS [20,21], and others. Colorimetric detection has advantages in terms of simplicity, cost-effectiveness, and sensitivity in visual monitoring [22,23]. Most colorimetric methods used for H_2_O_2_ detection are based on the color reaction of substrate 3, 3, 5, 5,—tetramethylbenzidine (TMB) [22,24,25] and o-phenylenediamine (OPD) [26,27,28] oxidized by H_2_O_2_ under the action of a catalyst to quantify H_2_O_2_. However, these organic compounds are toxic and are easily oxidized by O_2_. Therefore, developing a stable H_2_O_2_ detection method without chromogenic substrates is meaningful. Precious metal nanostructures have many excellent properties, such as good light scattering, absorption, and photothermal conversion capabilities [11,29]. Au NS is an uncommon type of gold nanostructure. In 2006, Nehl et al. [30] systematically studied gold nanostructures and multi-branched nanoparticles with sharp tips and unique local surface plasmon resonance (LSPR) characteristics. Au NS is a promising choice for biosensor application due to its sharp corners, plasma resonance, and lightning rod effect [31,32]. Research has shown that the LSPR characteristics of Au NS are related to their size, shape, and composition. Chemical etching can change the length of the sharp angle of Au NS and the surface plasmon resonance of Au NS, which means that the chemical etching of the sharp angle of Au NS will shift the LSPR absorption peak of Au NS and produce a blue color change [12,13,33]. The etching process can be adjusted by iodide to quickly and sensitively generate the blue shift of the surface plasmon resonance spectrum [34].

**Scheme 1:**
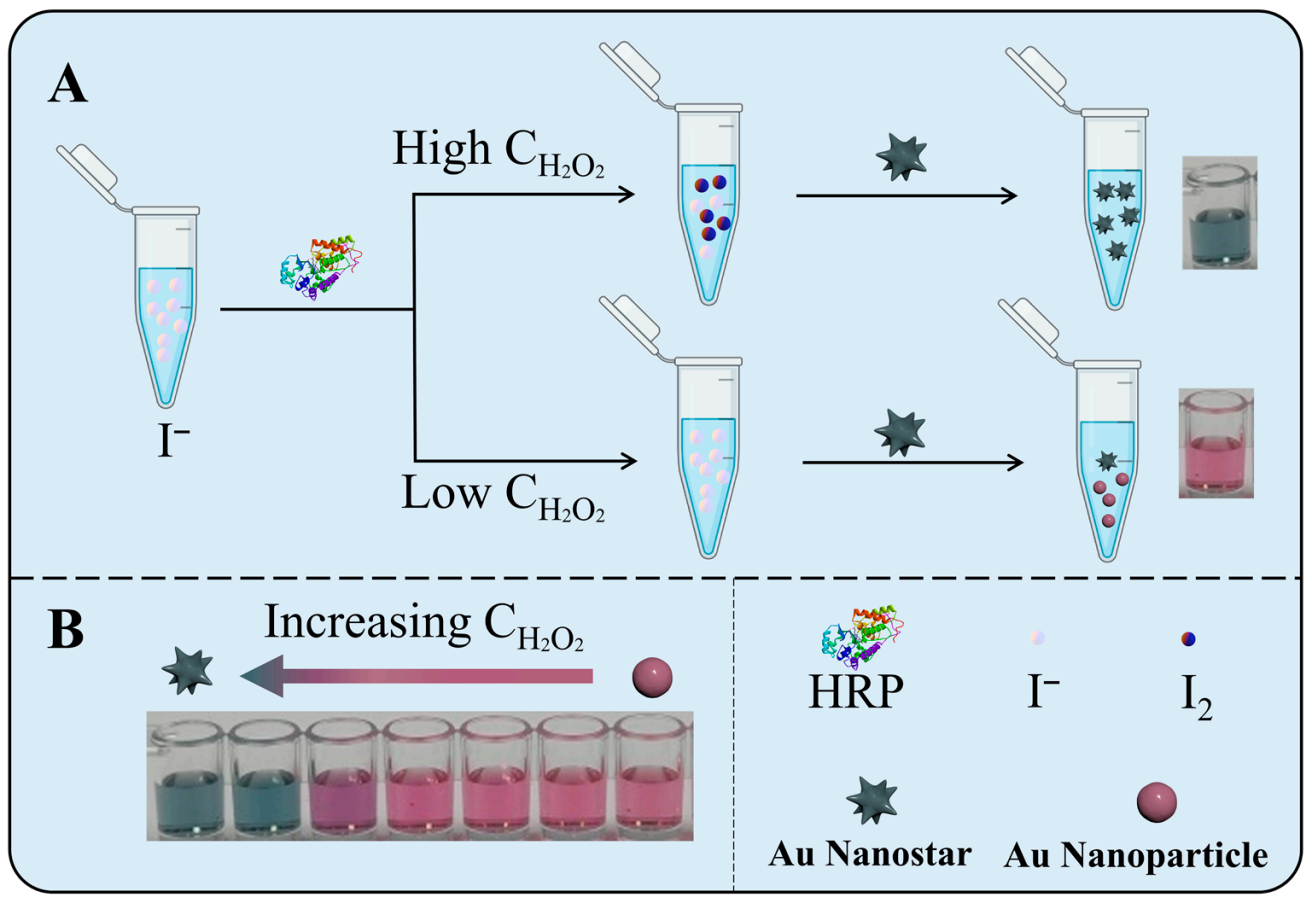
Schematic illustration of iodide-mediated etching of Au NS for H_2_O_2_ detection (**A**) and color changes with increasing H_2_O_2_ (**B**).

Considering the important role of H_2_O_2_, this work intended to construct a multicolor sensor based on the reaction between H_2_O_2_, Au NS, and I^−^ (Scheme 1A). Firstly, H_2_O_2_ reacts with I^−^ to generate I_2_ with the help of horseradish peroxidase (HRP). The remaining I^−^ etches the sharp corners of Au NS, which changes the absorption peak of LSPR shifts and the color of the solution. With the increasing concentration of I^−^, the degree of blue shift (Δλ) increases. The color of the mixture changes from blue to purple and finally turns pink, and Δλ can be used for the quantification of H_2_O_2_ (Scheme 1B).

## 2. Experimental Section

### 2.1. Reagents and Materials

Tetrachloroauric acid (HAuCl_4_) and trisodium citrate dihydrate (Na_3_C_6_H_5_O_7_∙2H_2_O) were purchased from Xilong Chemical Co., Ltd., Shantou, China; potassium iodide (KI) was purchased from Sinopharm Chemical Reagent Co., Ltd., Shanghai, China; H_2_O_2_, potassium chloride (KCl), potassium sulfate (K_2_SO_4_), and sodium nitrate (KNO_3_) were purchased from Xilong Science Co., Ltd., Shantou, China; 4-(2-Hydroxyethyl)Piperazine-1-ethanesulfonic acid sodium salt (HEPES-Na), horseradish peroxidase (HRP), tris(hydroxymethyl) aminomethane (Tris), and sodium carbonate (Na_2_CO_3_) were purchased from Aladdin Co., Ltd., Fukuoka, Japan. All chemicals are analytical grade, and the solution is prepared using deionized water obtained from the Milli-Q water purification system (≥18.2 M Ω cm^−1^, Milli-Q, Millipore, Burlington, MA, USA).

### 2.2. Apparatus

The transmission electron microscope (FE-TEM) images of Au NS were collected by JEOL200 kV field emission transmission electron microscope (JEM-2100 F, JEOL, Tokyo, Japan). We used KQ5200DE ultrasonic cleaner (Kunshan Ultrasonic Instrument Co., Ltd., Kunshan, China) to perform ultrasonic treatment on the sample and the UV–Vis spectrum was recorded by using a Tecan Spark microplate reader (Tecan, Switzerland).

### 2.3. Synthesis of Au NS [34]

Golden Seed: Add a trisodium citrate solution (4 mL, 1%) to a rapidly stirred boiling HAuCl_4_ aqueous solution (50 mL, 0.5 mmol L^−1^) and boil for 30 min. The solution color changes from purple-red to wine-red. Then, cool the wine-red solution to room temperature to obtain the gold seed solution, and store it at 4 ℃ for later use.

Au NS: Take 750 μL seeds solution, vigorously stir and add 38.5 mL of water, 18.75 mL of HEPES-Na (100 mmol L^−1^, pH = 8.5), and 750 μL hydroxylamine hydrochloride (HONH_2_HCl, 40 mmol L^−1^), then 22.5 mL of HAuCl_4_ (1 mmol L^−1^) was added to the above solution, and stir for 15 min to obtain a blue solution.

### 2.4. Detection of H_2_O_2_

After incubation at 37 ℃ for 40 min, 50 μL HRP (40 ng mL^−1^), 100 μL KI and 100 μL H_2_O_2_ of different concentrations were added into 250 μL Tris-HCl (10 mmol L^−1^, pH = 3.0) buffer solution. Take 50 μL and add it to 250 μL Au NS. After the reaction at 37 ℃ for 25 min, the ultraviolet absorption spectra of each pore were recorded by a Tecan microplate reader. The calibration curve of Δλ and H_2_O_2_ concentration was constructed by drawing the wavelength shift change values (Δλ) of different concentrations of H_2_O_2_, where wavelength change value Δλ = λ_0_ − λ (λ_0_ is the peak value of Au NS, λ is the peak value measured after the etching of Au NS).

### 2.5. Detection of H_2_O_2_ in Tap Water

A standard addition method was used to evaluate the feasibility of iodide-mediated Au NS etching for detecting H_2_O_2_ in tap water. Follow the above steps (drawing a standard curve) to determine the concentration of H_2_O_2_ in tap water by recording the change in wavelength offset (Δλ).

## 3. Results and Discussion

### 3.1. Characterization of AuNS

**Figure 1:**
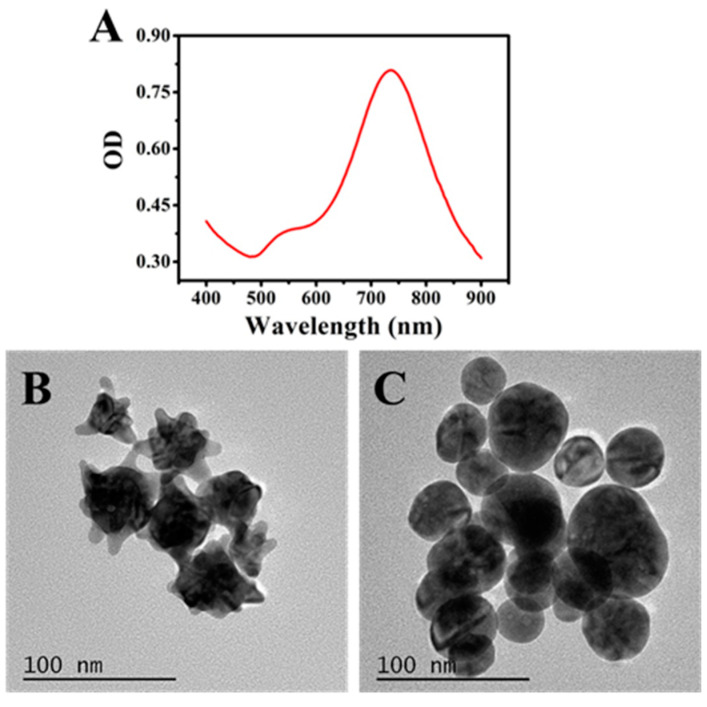
(**A**) UV–Vis spectrum of AuNS; (**B**) TEM images of AuNS before etching and (**C**) after etching.

AuNS are synthesized by a seed-mediated method. First, small gold nanoparticles are synthesized, and then gold seeds are added to the growth solution containing HAuCl_4_, a reducing agent, and surfactant. The newly reduced Au° grows on the surface of the seeds to form Au NS [18,35,36]. The UV–Vis spectrum measured using a Tecan microplate reader is shown in Figure 1A. The absorbance intensities of the Au NS solution at different wavelengths were recorded as the optical density (OD). It can be seen that the Au NS has two SRP bands located at 736 nm and 550 nm, respectively. λ_0_ represents the maximum absorption peak of Au NS at 736 nm. Figure 1B shows that Au NS has multiple sharp edges. Compared to smooth surfaces, the multiple sharp edges of Au NS have special sensitivity to the etching of I^−^, and iodide binds to the surface during the etching process [36]. After I^−^ etched Au NS, the sharp corners become shorter, and Au NS are etched into spherical nanostructures (Figure 1C). Due to the fact that gold atoms are “soft” Lewis acids, and I^−^ is a classic “soft” Lewis donor [34], iodide has a high affinity for gold. Oxidized Au ions and iodide can chelate at the interface of Au NS [37], resulting in uneven or even larger Au NS sizes after etching.

### 3.2. Feasibility of Iodide-Mediated Etching of AuNS for Detecting H_2_O_2_

**Figure 2:**
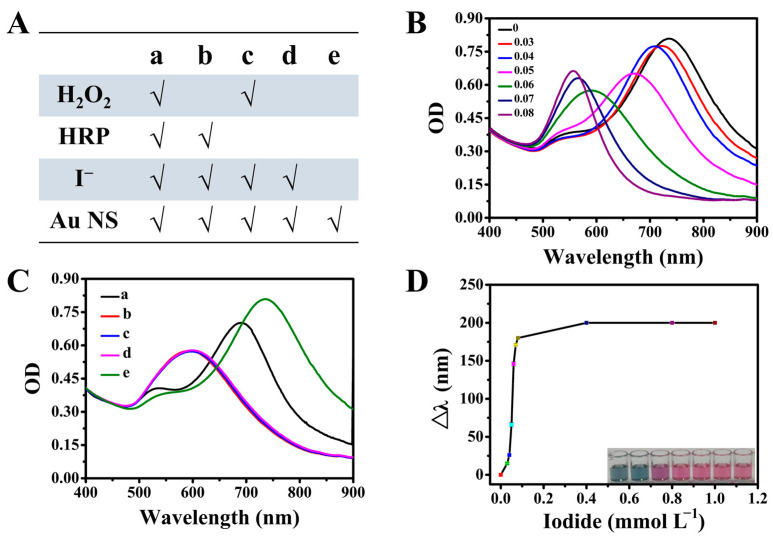
(**A**) Table of the solutions (a, b, c, d, e) with different components for the etching of AuNS. (**B**) The corresponding UV−Vis spectra of AuNS after etching with different components. (**C**) UV−Vis spectra of AuNS after etching by different concentrations of I^−^. (**D**) The relationship between etching degree and I^−^ concentration. Inset is the color change with varying degrees of etching.

To investigate the feasibility of using iodide-mediated surface etching of AuNS for detecting H_2_O_2_, we first validated the UV–Vis spectra of different mixtures. As shown in Figure 2A,B, when only Au NS exists, the absorption peak was measured at 736 nm (Figure 2B, curve e). After adding a final concentration of I^−^ as 80 μmol L^−1^, the peak of the UV visible absorption peak changed from 736 nm to 588 nm, resulting in a significant blue shift of the UV peak absorption peak (Figure 2B, curve d). The addition of H_2_O_2_ and HRP separately does not affect the etching of AuNS by I^−^ (curves b and c in Figure 2B). Only when both exist simultaneously does the absorption peak of the mixture shift to red (curve as in Figure 2B). This is because the addition of H_2_O_2_ or HRP alone into the reaction system does not affect the concentration of I^−^, and all I^−^ is used for etching AuNS. After the addition of both, HRP can catalyze the oxidation of iodide to elemental iodine in the presence of H_2_O_2_ [34]. It led to a decrease in the remaining concentration of iodide. A decrease etching degree of Au NS was also observed which resulted in a red shift of the absorption peak of Au NS. In addition, we validated the relationship between the concentration of I^−^ and the degree of etching. When the concentration of I^−^ increases from 0 mmol L^−1^ to 1.2 mmol L^−1^, the etching degree gradually increases and reaches the plateau (Figure 2D), and the corresponding absorption peak shifts from 736 nm to 556 nm. At the same time, the color of the solution changes from dark blue to purple to pink (Inset of Figure 2D). In Figure 2C, it can be seen that as the concentration of I^−^ increases from 0 mmol L^−1^ to 0.08 mmol L^−1^, the etching degree gradually increases. When the concentration of I^−^ increases from 0.05 mmol L^−1^ to 0.06 mmol L^−1^, the maximum absorption peak of the Au NS solution shows a large blue shift (Δλ). However, the increase in Δλ becomes little from 0.06 mmol L^−1^ to 0.08 mmol L^−1^. Therefore, an I^−^ concentration of 0.06 mmol L^−1^ was selected for the sensitive detection of H_2_O_2_. The above experimental results indicate that this method based on the iodide-mediated etching of AuNS for detecting H_2_O_2_ has good feasibility.

### 3.3. Optimization of Experimental Conditions

**Figure 3:**
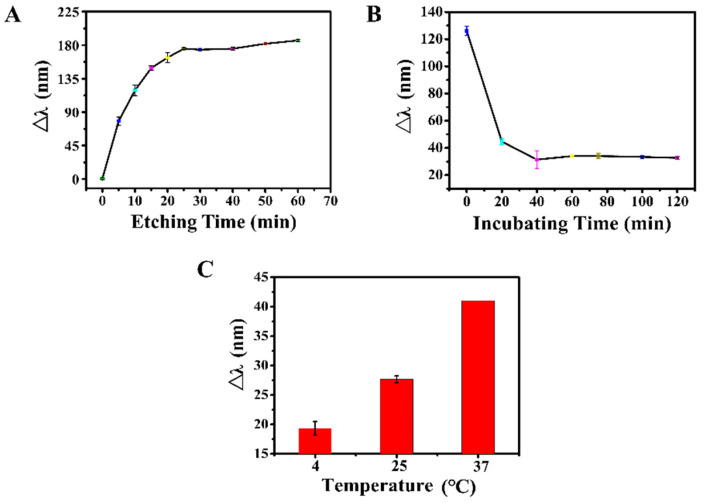
Optimization of (**A**) time for I^−^ etching of AuNS at 37 °C; (**B**) response time for the reaction between HRP and H_2_O_2_; (**C**) reaction temperature.

To obtain a more sensitive response, several parameters in the experimental system were optimized, such as the time for I^−^ etching AuNS, the incubation time for the reaction between HRP and H_2_O_2_, and the reaction temperature (Figure 3). As shown in Figure 3A, the reaction time for I^−^-induced etching of AuNS was optimized. After adding a concentration of 0.06 mmol L^−1^, the plateau was reached with a reaction time of 25 min, indicating that the etching of AuNS by I^−^ can reach stability after 25 min. Figure 3B shows the optimization of incubation time for HRP and H_2_O_2_. At this point, the concentration of H_2_O_2_ is 33.3 μmol L^−1^. As the time of HRP catalyzing H_2_O_2_ prolongs, the etching degree gradually decreases and reaches stability. This is because the amount of I^−^ consumed by H_2_O_2_ under HRP catalysis increases, resulting in a decrease in the remaining I^−^, leading to a decrease in the degree of Au NS etching and a shift in the UV absorption peak. Finally, we optimized the reaction temperature. The experimental results indicate that an increase in temperature contributes to the etching of the reaction system (Figure 3C), but a higher temperature will reduce the catalytic efficiency of HRP. Therefore, we chose a reaction time of 25 min for I^−^-induced etching of AuNS, an incubation time of 40 min for the reaction between HRP and H_2_O_2_, and a reaction temperature of 37 ℃.

### 3.4. Performance Analysis of Iodide-Mediated AuNS Etching for Detecting H_2_O_2_

**Figure 4:**
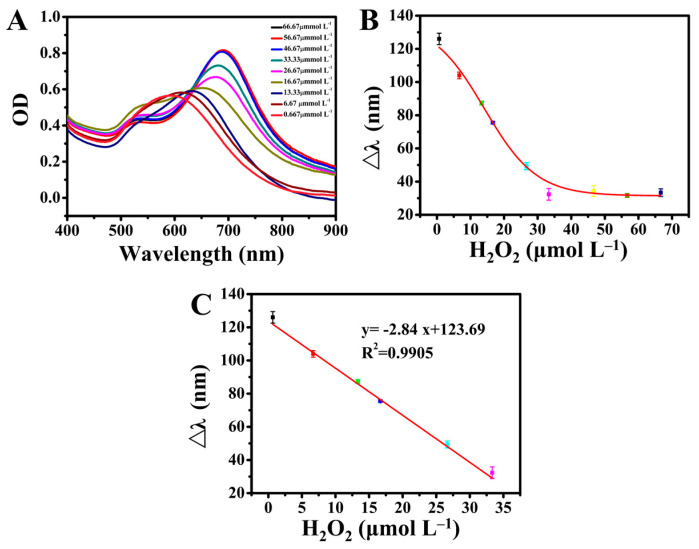
(**A**) UV–Vis spectra of iodide-mediated etching of AuNS in the presence of H_2_O_2_ with different concentrations. (**B**) The responses of Δλ to different concentrations of H_2_O_2_. (**C**) Linear relationship between Δλ and H_2_O_2_ concentration.

Under optimal experimental conditions, the linear response range and detection limit of H_2_O_2_ detected by the constructed sensor platform were investigated by monitoring the offset value of the absorption peak (Δλ). As shown in Figure 4A,B, with the increase in H_2_O_2_ concentration in the range of 0.67–66.67 μmol L^−1^, Δλ gradually increases and reaches a plateau. In the range of 0.67–33.33 μmol L^−1^, Δλ has a strong linear relationship with the concentration of H_2_O_2_ (Figure 4C). The linear equation is Δλ = −2.84C_H_2_O_2__ + 123.69 (R^2^ = 0.9908, *n* = 3) and the LOD value is 0.44 μmol L^−1^ (*n* = 20, 3σ_0_/K). Compared with other reported H_2_O_2_ sensors (Table S1), this method is rapid and sensitive, and shows a multicolor change which has great potential application in the field of point-of-care testing.

### 3.5. The Selectivity and Interference Study of Iodide-Mediated Etching of AuNS for Detecting H_2_O_2_

**Figure 5:**
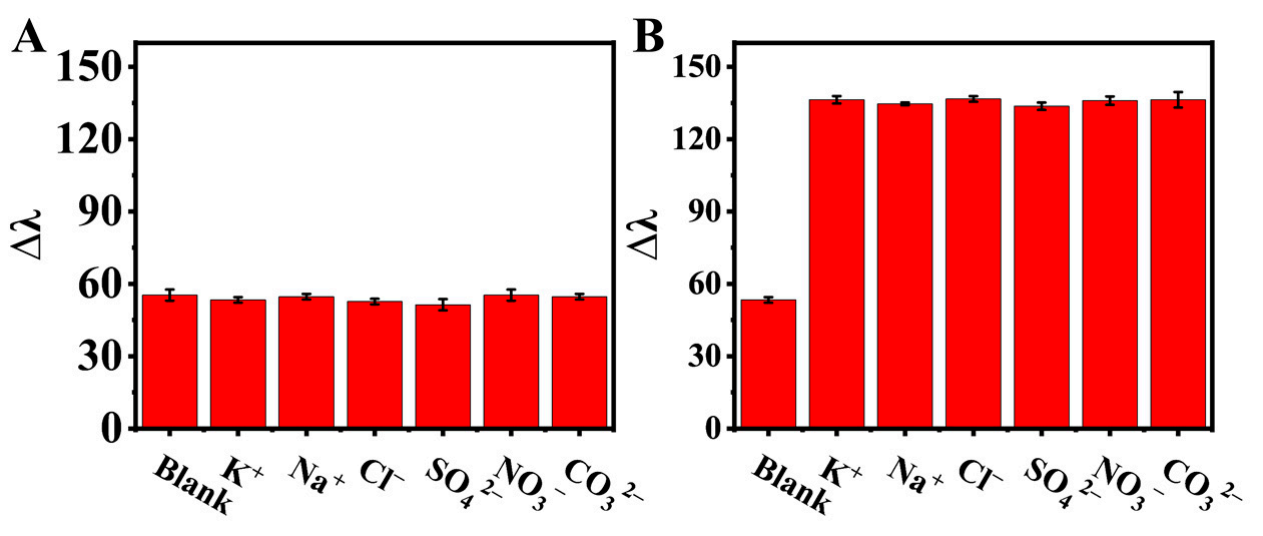
(**A**) The effect of (K^+^, Na^+^, Cl^−^, NO_3_^−^, SO_4_^2−^, CO_3_^2−^) on the selectivity detection of H_2_O_2_. The concentration of H_2_O_2_ was 26.67 μmol L^−1^. (**B**) The interference study for detecting H_2_O_2_.

Tap water often contains a variety of salts, so it is necessary to explore the interference of different ions in the detection of hydrogen peroxide in tap water. To evaluate the selectivity and interference study of this method for H_2_O_2_ detection in tap water, six different interfering substances (K^+^, Na^+^, Cl^−^, NO_3_^−^, SO_4_^2−^, CO_3_^2−^,) were selected for the study. Figure 5A shows that under the same experimental conditions, except for H_2_O_2_, even if the concentration of other interfering substances is 50 times higher than the concentration of H_2_O_2_, the Δλ shows no significant decrease. The above results indicate that this method has high selectivity for the detection of H_2_O_2_. Subsequently, we selected the above-mentioned interfering substances for the interference study. As shown in Figure 5B, the Δλ value of H_2_O_2_ detection with the addition of interfering substances did not change. The results indicate that this method has good stability for detecting H_2_O_2_.

### 3.6. Detection of H_2_O_2_ in Real Samples

To evaluate the potential applicability of this method for detecting H_2_O_2_ in real samples, tap water was selected as the actual sample, and the accuracy of the method was verified by the spiked recovery method. The specific experimental steps are as follows: Take 30% H_2_O_2_ and dilute it with tap water to different concentrations. Add 50 μL HRP (40 ng mL^−1^) and 100 μL NaI to Tris-HCl (10 mmol L^−1^, pH = 3.0) buffer solution. After incubating with 100 μL different concentrations of H_2_O_2_ at 37 °C for 40 min, 50 μL of the above solution was added to 250 μL Au NS, and the reaction was carried out at 37 °C for 25 min. UV measurement was performed after the reaction was completed. Unless otherwise specified, all detection experiments were independently repeated three times. Statistical analysis was conducted on the data obtained from at least three independent experiments. The data of all detection methods are represented as mean ± standard deviation (SD). The recovery rate of this method is between 92.8–101.6%, and the relative standard deviation (RSD) is between 3.4–4.4% (Table 1). These results indicated the superior accuracy and potential application of this method based on the iodide-mediated etching of AuNS for detecting H_2_O_2_.

## 4. Conclusions

In this work, AuNS was utilized to construct a multicolor sensor for detecting H_2_O_2_ based on a rapid and sensitive surface etching of Au NS. AuNS are etched into spherical nanostructures in the presence of HRP, I^−^, and H_2_O_2_. The etching process also produces color changes with different concentrations of H_2_O_2_ which can be used for visual detection. This method exhibits satisfactory sensitivity and selectivity. Under the conditions of an incubation time of 40 min, a reaction time of 25 min, a temperature of 37 °C, and I^−^ concentration of 0.06 mmol L^−1^, the blue shift value of the absorption peak ∆λ shows a good linear relationship with the concentration of H_2_O_2_, with a linear range of 0.667~66.67 μmol L^−1^, and a detection limit of 0.44 μmol L^−1^. Because of the usage of the biological enzyme of HRP in this work, it is important to control the temperature during the analysis. This shortcoming might be solved by using a nanozyme to replace HRP. Considering the significant role of H_2_O_2_ in food chemistry and biochemistry, this work offered a new multicolor visual method for point-of-care testing and on-site analysis.

## Figures and Tables

**Table 1:** Detection of H_2_O_2_ in tap water samples.

Sample Number	Added (μmol L^−1^)	Found (μmol L^−1^)	Recovery (%)	RSD (*n* = 3, %)
1	10.0	9.28 ± 0.41	92.8	4.4
2	20.0	20.31 ± 0.70	101.6	3.5
3	30.0	28.18 ± 0.96	93.9	3.4

## Data Availability

Not applicable.

## Supplementary Materials

The following supporting information can be downloaded at: https://www.mdpi.com/article/10.3390/bios13060585/s1, Table S1: comparative analysis of this method with other colorimetric methods for detecting H_2_O_2_. References [38,39,40,41,42,43,44] are cited in the Supplementary Materials.
