# Supplementary material for: Iodide-Mediated Etching of Gold Nanostar for the Multicolor Visual Detection of Hydrogen Peroxide
Source: Biosensors (Basel). 2023 May 28;13(6):585. doi: 10.3390/bios13060585 (PMC10296679; doi:10.3390/bios13060585)
Supplement: Supplementary file 1 [file biosensors-13-00585-s001.zip › biosensors-2389680-supplementary.pdf]

biosensors

MDPI

Supplementary Material

Iodide-Mediated Etching of Gold Nanostar for the Multicolor Visual Detection of Hydrogen Peroxide

Yunping Lai [1,†], Beirong Yu [1,†], Tianran Lin [1,*] and Li Hou[1,*]

1 School of Chemistry and Pharmaceutical Sciences, State Key Laboratory for the Chemistry and Molecular Engineering of Medicinal Resources, Guangxi Normal University, Guilin 541004, China; lyp159357@stu.gxnu.edu.cn (Y.L.); yubeirong@stu.gxnu.edu.cn (B.Y.);

* Correspondence: tianran@gxnu.edu.cn (T.L.); houli@mailbox.gxnu.edu.cn (L.H.)

† These authors contributed equally to this paper.

Table S1. Comparative analysis of this method with other colorimetric methods for detecting H_2O_2.

Method	Recognization/Sensing elements	Substrate	Color changes	Limit of detection (μmmol L^{-1})	Reference
Colorimetric	MFe_2O_4	OPD	Single color	30	[1]
Colorimetric	$MnCo_2O_4$	OPD	Single color	200	[2]
Colorimetric	AuAgPd TNPs	OPD	Single color	5.6	[3]
Colorimetric	Fe, N-CD	OPD	Single color	0.05	[4]
Colorimetric	PtNWs-PNFs/GO	TMB	Single color	0.0206	[5]
Colorimetric	Fe-NDs	TMB	Single color	0.3	[6]
Colorimetric	Fe-C-NNs	TMB	Single color	4.36	[7]
Colorimetric	$Co_3(PO_4)\cdot 8H_2O$	TMB	Single color	4.385	[8]
Colorimetric	Au NS	Au NS	Multicolor	0.44	This work

References

1. Vetr, F.; Moradi-Shoeili, Z.; Özkar, S. Oxidation of o-phenylenediamine to 2,3-diaminophenazine in the presence of cubic ferrites MFe_2O_4 (M = Mn, Co, Ni, Zn) and the application in colorimetric detection of H_2O_2. *Appl Organomet Chem* **2018**, *32*, e4465, doi:10.1002/aoc.4465.
2. Vetr, F.; Moradi-Shoeili, Z.; Özkar, S. Mesoporous $MnCo_2O_4$ with efficient peroxidase mimetic activity for detection of H_2O_2. *Inorg. Chem Commun* **2018**, *98*, 184-191, doi:10.1016/j.inoche.2018.10.024.
3. Kong, J.; Zheng, J.; Li, Z.; Huang, J.; Cao, F.; Zeng, Q.; Li, F. One-pot synthesis of AuAgPd trimetallic nanoparticles with peroxidase-like activity for colorimetric assays. *Anal Bioanal Chem* **2021**, *413*, 5383-5393, doi:10.1007/s00216-021-03514-1.
4. Wang, L.; Liu, Y.; Yang, Z.; Wang, Y.; Rao, H.; Yue, G.; Wu, C.; Lu, C.; Wang, X. A ratiometric fluorescence and colorimetric dual-mode assay for H_2O_2 and xanthine based on Fe, N co-doped carbon dots. *Dyes Pigm* **2020**, *180*, 108486, doi:10.1016/j.dyepig.2020.108486.
5. Zhu, D.; He, P.; Kong, H.; Yang, G.; Luan, X.; Wei, G. Biomimetic graphene-supported ultrafine platinum nanowires for colorimetric and electrochemical detection of hydrogen peroxide. *J Mater Chem B* **2022**, *10*, 9216-9225, doi:10.1039/d2tb02132c.
6. Liu, Y.; Yan, J.; Huang, Y.; Sun, Z.; Zhang, H.; Fu, L.; Li, X.; Jin, Y. Single-Atom Fe-Anchored Nano-Diamond With Enhanced Dual-Enzyme Mimicking Performance for H_2O_2 and Glutathione Detection. *Front Bioeng Biotechnol* **2021**, *9*, 790849, doi:10.3389/fbioe.2021.790849.
7. Lu, W.; Chen, S.; Zhang, H.; Qiu, J.; Liu, X. Fe N C single atom nanozymes with dual enzyme-mimicking activities for colorimetric detection of hydrogen peroxide and glutathione. *J Materiomics* **2022**, *8*, 1251-1259, doi:10.1016/j.jmat.2022.04.011.
8. Peng, L.-J.; Zhou, H.-Y.; Zhang, C.-Y.; Yang, F.-Q. Study on the peroxidase-like activity of cobalt phosphate and its application in colorimetric detection of hydrogen peroxide. *Colloids Surf A Physicochem Eng Asp* **2022**, *647*, 129031, doi:10.1016/j.colsurfa.2022.129031.
